# MicroRNA-520g promotes epithelial ovarian cancer progression and chemoresistance via DAPK2 repression

**DOI:** 10.18632/oncotarget.8530

**Published:** 2016-04-01

**Authors:** Jing Zhang, Lei Liu, Yunyan Sun, Jiandong Xiang, Dongmei Zhou, Li Wang, Huali Xu, Xiaoming Yang, Na Du, Meng Zhang, Qin Yan, Xiaowei Xi

**Affiliations:** ^1^ Department of Obstetrics and Gynecology, Shanghai Jiao Tong University Affiliated First People's Hospital, Shanghai, China; ^2^ Department of Obstetrics and Gynecology, Ren Ji Hospital, School of Medicine, Shanghai Jiao Tong University, Shanghai, China; ^3^ Department of Pathology, Fudan University Affiliated Shanghai Cancer Center, Shanghai, China

**Keywords:** miR-520g, epithelial ovarian cancer, progression, chemoresistance, DAPK2

## Abstract

The lack of efficient tumor progression and chemoresistance indicators leads to high mortality in epithelial ovarian cancer (EOC) patients. Dysregulated miR-520g expression is involved in these processes in hepatic and colorectal cancers. In this study, we found that miR-520g expression gradually increased across normal, benign, borderline and EOC tissues. High miR-520g expression promoted tumor progression and chemoresistance to platinum-based chemotherapy, and reduced survival in EOC patients. miR-520g upregulation increased EOC cell proliferation, induced cell cycle transition and promoted cell invasion, while miR-520g downregulation inhibited tumor-related functions. *In vivo*, overexpression or downregulation of miR-520g respectively generated larger or smaller subcutaneous xenografts in nude mice. Death-associated protein kinase 2 (DAPK2) was a direct target of miR-520g. In 116 EOC tissue samples, miR-520g expression was significantly lower following DAPK2 overexpression. DAPK2 overexpression or miR-520g knockdown reduced EOC cell proliferation, invasion, wound healing and chemoresistance. This study suggests that miR-520g contributes to tumor progression and drug resistance by post-transcriptionally downregulating DAPK2, and that miR-520g may be a valuable therapeutic target in patients with EOC.

## INTRODUCTION

Most epithelial ovarian cancer (EOC) patients are diagnosed at advanced stages of disease due to an absence of early clinical symptoms and early diagnostic biomarkers, resulting in poor prognosis and short survival [1]. As a result, EOC is the most lethal gynecologic cancer worldwide [2]. Cytoreductive or radical surgery accompanied by platinum-based chemotherapy is usually recommended for advanced EOC [3]. However, the mean 5-year survival rate is still low at 46%, which is mainly attributable to chemoresistance and the aggressive nature of the disease [4]. Despite the availability of studies describing the molecular mechanisms underlying EOC tumor progression and chemoresistance, no reliable markers exist for prediction of chemoresistance [5].

Several studies reported that aberrant expression of microRNAs (miRNAs) played a significant role in chemoresistance and progression of EOC [6–8]. miRNAs are small non-coding RNAs (20–24 nucleotides) that exist stably in human serum and mediate biological processes such as cell proliferation, apoptosis and cell cycle regulation [9, 10]. miRNAs negatively regulate their targeted genes by specifically binding and degrading the 3′ untranslated regions (3′UTRs) of targeted genes [11]. Therefore, in this study miRNAs were divided into onco-miRNAs and suppressors according to their targeted gene function in tumors [12].

MiR-520g is a member of the miR-520 family, which includes tumor-promoting miRNAs [13]. In progesterone receptor and estrogen receptor-negative breast cancer patients, high miR-520g levels were negatively correlated with prognosis [14, 15]. In hepatocellular carcinoma, miR-520g upregulation promoted tumor cell invasion and metastasis by reducing SMAD7 expression and inducing epithelial–mesenchyme transition (EMT) [16]. Further, miR-520g increased chemoresistance to 5-fluorouracil (5-FU) by modulating P21 expression in colorectal cancer [17].

Death-associated protein kinase 2 (DAPK2) is a serine/threonine kinase that plays a positive role in cellular apoptosis and autophagy [18–20]. Targeted gene prediction showed that miR-520g directly recognized and bound the DAPK2 3′UTR. However, whether miR-520g induces tumor progression and chemoresistance by directly downreguating DAPK2 in EOC is unclear.

Herein, we demonstrated that miR-520g expression is significantly increased in EOC and high miR-520g expression promotes tumor development, increases chemoresistance to platinum-based chemotherapy and reduces patient survival. We also demonstrated that miR-520g directly targets and downregulates DAPK2 by binding the DAPK2 3′UTR. Finally, we showed that DAPK2 suppression, followed by MAPK and AKT pathway activation, promotes the biological processes mediated by miR-520g in EOC.

## RESULTS

### miR-520g upregulation promotes tumor progression and predicts poor survival in EOC patients

**Figure 1:**
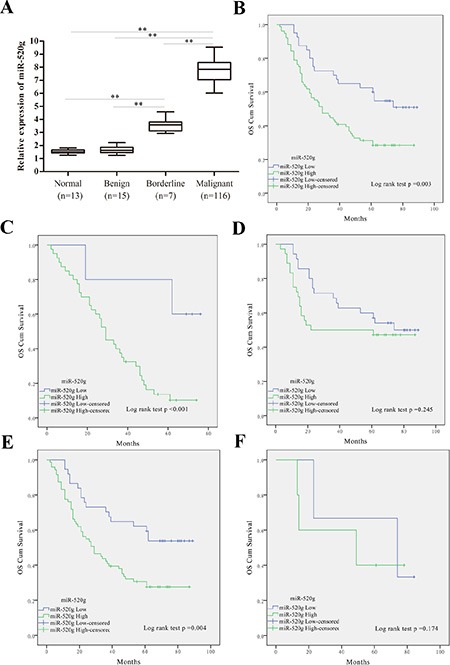
miR-520g was upregulated in EOC tissues and high miR-520g expression predicted poor EOC patient survival, especially in recurrent or high serum CA-125 level patients (**A**) Relative miR-520g expression in normal, benign, borderline and EOC tissues (**p < 0.001). (**B**) Estimation of overall survival curves by Kaplan–Meier analysis with log-rank test in 116 EOC patients according to miR-520g expression. Patients with high miR-520g expression worse overall survival than patients with low expression. (**C-F**) Overall survival curves obtained by Kaplan–Meier analysis with the log-rank test in patients with tumor recurrence (*n* = 45, C), no recurrence (*n* = 71, D), high serum CA-125 level (*n* = 108, E), and low serum CA-125 level (*n* = 8, F). High miR-520g expression indicated poorer patient survival with tumor recurrence or high serum CA-125 level.

We found that miR-520g expression gradually increased across 13 normal, 15 benign, 7 borderline and 116 EOC tissue samples, indicating that miR-520g upregulation may mediate the development of EOC (Figure 1A). Thirty of 110 patients with ovarian cancer were chemoresistant and 86 were sensitive to chemotherapy (Table 1). Ninety percent of the patients in the chemoresistant group, but only 57% of patients in the chemosensitive group, had high tissue expression of miR-520g (Table 1, *p* < 0.001).

**Table 1:** Clinicopathologic characteristics of 116 EOC patients

Characteristics	No. of patients (%)	Chemoresistance	
		No *n* = 86 (%)	Yes *n* = 30 (%)
Age			
< 50	43 (37.1)	29 (33.7)	14 (46.7)
≥ 50	73 (62.9)	57 (66.3)	16 (53.3)
Ascites			
< 100	41 (35.3)	35 (40.7)	6 (20.0)
≥ 100	75 (64.7)	51 (59.3)	24 (80.0)
Serum CA-125 level			
< 35	8 (7.0)	6 (7.0)	2 (6.7)
≥ 35	108 (93.0)	80 (93.0)	28 (93.3)
Lymph node metastasis			
Negative	71 (61.2)	71 (82.6)	0 (0.0)
Positive	45 (38.8)	15 (17.4)	30 (100.0)
Tumor differentiation			
G1	23 (19.8)	20 (23.3)	3 (10.0)
G2	41 (35.3)	34 (39.5)	7 (23.3)
G3	52 (44.8)	32 (37.2)	20 (66.7)
Histology type			
Serous	82 (70.7)	64 (74.3)	18 (70.7)
Mucinous	14 (12.1)	12 (14.0)	2 (12.1)
Endometrioid	16 (13.8)	9 (10.5)	7 (13.8)
Clear cell	4 (3.4)	1 (1.2)	3 (3.4)
Residual tumor size			
< 1 cm	80 (69.0)	67 (77.9)	13 (43.3)
≥ 1 cm	36 (31.0)	19 (22.1)	17 (56.7)
FIGO stage			
I–II	15 (12.9)	1 (15.1)	2 (6.7)
III–IV	101 (87.1)	73 (84.9)	28 (93.3)
Chemotherapy plan			
TP	73 (62.9)	51 (59.3)	22 (73.3)
PAC	43 (37.1)	35 (40.7)	8 (26.7)
miR-520g expression			
Low	40 (34.5)	37 (43.0)	3 (10.0)
High	76 (65.5)	49 (57.0)	27 (90.0)

FIGO: International Federation of Gynecology and Obstetrics.

We further analyzed correlations between clinical features and miR-520g expression in the 116 EOC patients. We found that 65.5% (76/116) of the patients showed a high miR-520g expression (Table 2). miR-520g upregulation was positively correlated with ascites (*p* = 0.017), lymph node metastasis (*p* < 0.001), tumor differentiation (*p* = 0.002), residual tumor size (*p* < 0.001), FIGO (International Federation of Gynecology and Obstetrics) stage (*p* < 0.001), chemotherapy regimen (*p* = 0.004) and chemoresistance (*p* < 0.001), while no correlations were observed with respect to patient age (*p* = 0.738), serum CA-125 level (*p* = 0.852) and histology type (*p* = 0.114) (Table 2).

**Table 2:** Correlations between clinical features and miR-520g expression in 116 ovarian cancer patients

Characteristics	No. of patients (%)	miR-520g expression		*P* *
		Low *n* = 40 (%)	High *n* = 76 (%)	
Age				
< 50	43 (37.1)	14 (35.0)	29 (38.2)	.738
≥ 50	73 (62.9)	26 (65.0)	47 (61.8)	
Ascites				
< 100	41 (35.3)	20 (50.0)	21 (27.6)	0.017
≥ 100	75 (64.7)	20 (50.0)	55 (72.4)	
Serum CA-125 level				
< 35	8 (7.0)	3 (7.5)	5 (6.6)	0.852
≥ 35	108 (93.0)	37 (92.5)	31 (93.4)	
Lymph node metastasis				
Negative	71 (61.2)	35 (82.6)	36 (0.0)	< 0.001
Positive	45 (38.8)	5 (17.4)	40 (100.0)	
Differentiation				
G1	23 (19.8)	12 (30.0)	11 (14.5)	0.002
G2	41 (35.4)	19 (47.5)	22 (28.9)	
G3	52 (44.8)	9 (22.5)	43 (56.6)	
Histology type				
Serous	82 (70.7)	28 (70.0)	54 (71.1)	0.114
Mucinous	14 (12.1)	8 (20.0)	6 (7.9)	
Endometrioid	16 (13.8)	4 (10.0)	12 (15.8)	
Clear cell	4 (3.4)	0 (0.00)	4 (5.3)	
Residual tumor size				
< 1 cm	80 (69.0)	37 (92.5)	43 (56.6)	< 0.001
≥ 1 cm	36 (31.0)	3 (7.5)	33 (43.4)	
FIGO stage				
I–II	15 (12.9)	12 (30.0)	3 (3.9)	< 0.001
III–IV	101 (87.1)	28 (70.0)	73 (96.1)	
Chemotherapy plan				
TP	73 (62.9)	18 (45.0)	55 (72.3)	0.004
PAC	43 (37.1)	22 (55.0)	21 (27.7)	
Chemoresistance				
Yes	30 (25.9)	2 (5.0)	28 (32.6)	< 0.001
No	86 (74.1)	38 (95.0)	48 (67.4)	

* *p* < 0.05 indicates a significant relationship among the variables.

FIGO: International Federation of Gynecology and Obstetrics.

TP: cisplatin and paclitaxel, PAC: cisplatin, epirubicin, and cyclophosphamide.

Subsequent Kaplan–Meier analysis with log-rank test revealed lower overall survival (OS) rates in patients with miR-520g upregulation as compared to patients with low tumor miR-520g expression (*p* = 0.003, Figure 1B). These results indicate that miR-520g is a prognostic marker in EOC patients. Furthermore, miR-520g expression was negatively associated with OS rates in patients with tumor recurrence (*p* < 0.001, Figure 1C) or high serum CA-125 levels (*p* = 0.004, Figure 1E). However, no differences were observed between miR-520g expression and OS rates in patients without tumor recurrence (*p* = 0.245, Figure 1D) or with low serum CA-125 levels (*p* = 0.174, Figure 1F). These results suggest that miR-520g predicts survival in EOC patients with tumor recurrence or high serum CA-125 levels. Univariate and multivariate Cox proportional hazards models showed that high miR-520g expression was independently associated with EOC progression (Table 3).

**Table 3:** Univariate and multivariate Cox proportional hazard models for overall survival (OS) and progression-free survival (PFS) in all EOC patients

	OS				PFS			
	Univariate		Multivariate		Univariate		Multivariate	
	HR (95% CI)	p*	HR (95% CI)	p*	HR (95% CI)	p*	HR (95% CI)	p*
miR-520g								
Low	-		-		-		-	
High	5.41 (3.71, 9.06)	< 0.001*	6.16 (3.15, 11.01)	0.003*	5.32 (2.03, 9.08)	< 0.001*	4.05 (1.43, 11.14)	0.036*
Age								
< 50	-		-		-		-	
≥ 50	1.76 (0.98, 4.35)	0.436			1.17 (0.89, 2.43)	0.691		
Ascites								
< 100	-		-		-		-	
≥ 100	1.36 (0.83, 2.28)	0.076			0.94 (0.79, 1.86)	0.447		
Serum CA-125 level								
< 35	-		-		-		-	
≥ 35	1.59 (1.04, 1.94)	0.654			2.36 (1.76, 3.89)	0.271		
Lymph node metastasis								
Negative	-		-		-		-	
Positive	5.34 (3.75, 10.01)	0.023*	4.17 (1.68, 9.78)	0.015*	3.41 (1.57, 11.14)	0.016*	3.45 (1.24, 7.52)	0.007*
Tumor differentiation								
G1	-		-		-		-	
G2	4.53 (1.67, 8.52)	0.043*	3.03 (0.58, 8.02)	0.071	2.51 (1.82, 5.77)	0.041*	1.40 (0.72, 3.70)	0.032*
G3	3.98 (2.79, 10.01)	< 0.001*	2.56 (1.35, 9.01)	0.031*	5.65 (1.44, 10.32)	0.028*	2.46 (1.63, 6.47)	< 0.001*
Histology type								
Serous	-		-		-		-	
Mucinous	4.14 (2.72, 9.59)	0.667			4.03 (2.15, 6.78)	0.534		
Endometrioid	3.12 (1.17, 6.78)	0.335			2.97 (1.56, 5.74)	0.721		
Clear cell	2.01 (1.48, 4.91)	0.218			2.67 (1.83, 5.46)	0.054		
Residual tumor size								
< 1 cm	-		-		-		-	
≥ 1 cm	4.02 (2.97, 8.41)	0.014*	2.31 (1.52, 5.43)	< 0.001*	4.21 (2.48, 8.97)	0.027*	3.11 (1.68–8.45)	0.047*
FIGO stage								
I–II	-		-		-		-	
III–IV	3.59 (2.96, 9.15)	0.021*	2.41 (1.38, 5.17)	0.007*	4.98 (3.37, 6.46)	0.025*	3.56 (2.05, 11.21)	< 0.001
Chemotherapy								
TP	-				-			
PAC	1.34 (0.97–2.85)	0.448			1.37 (1.02–1.91)	0.524		0.067

HR, hazard ratio; CI, confidence interval.

* *p* < 0.05 indicated that the 95% CI of HR was not including 1.

FIGO: International Federation of Gynecology and Obstetrics.

TP: cisplatin and paclitaxel, PAC: cisplatin, epirubicin, and cyclophosphamide.

### miR-520g promotes proliferation, cell cycle progression, invasion and chemoresistance in EOC cells

**Figure 2:**
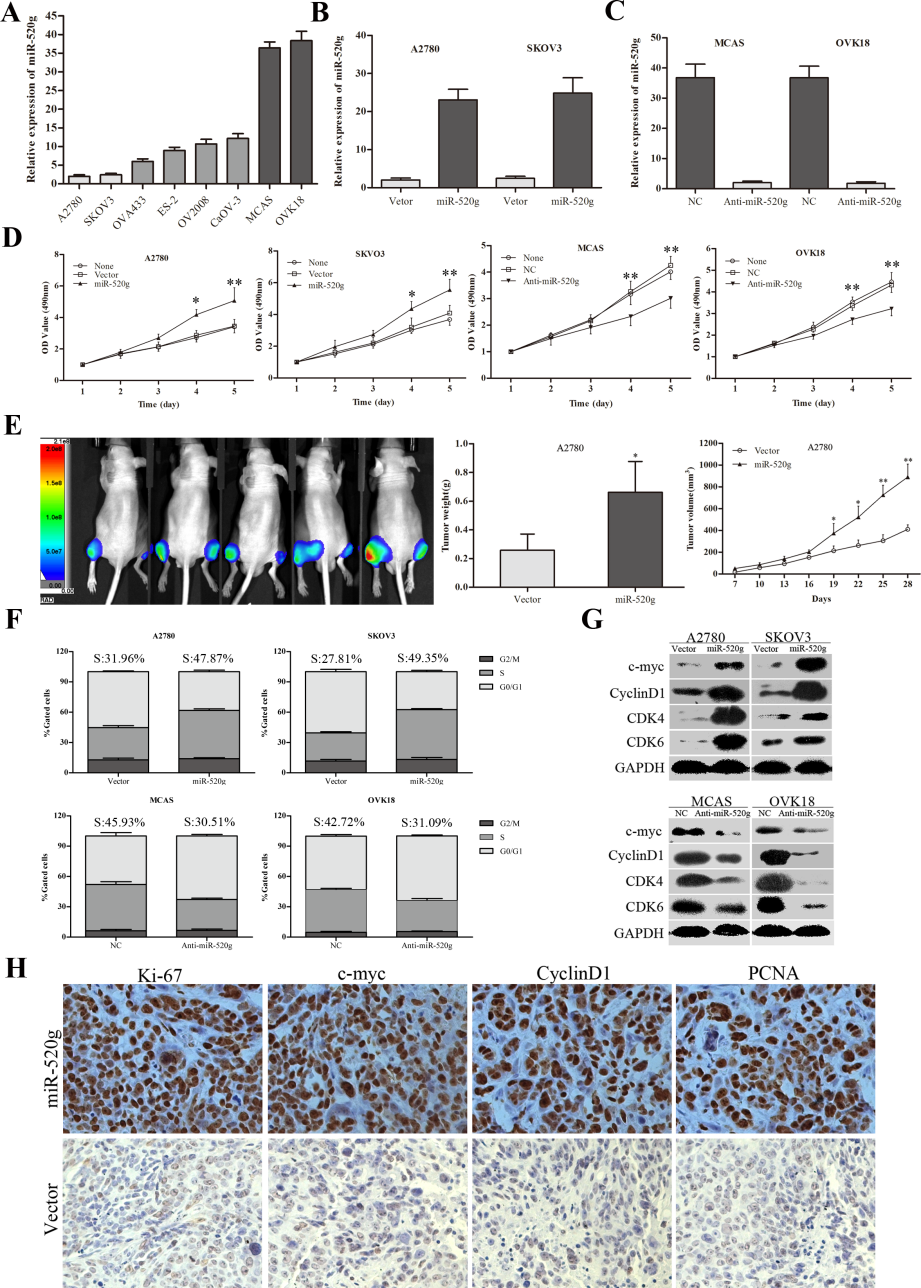
miR-520g promoted EOC cell proliferation and cell cycle transition *in vitro* and *in vivo* (**A**) Relative miR-520g expression in eight EOC cell lines. (**B**) miR-520g was upregulated after transfection with miR-520g overexpression vector. (**C**) miR-520g was downregulated after transfection with anti-miR-520g vector. (**D**) miR-520g overexpression or knockdown resulted in increased or decreased EOC cell proliferation, respectively, *in vitro*. (**E**) Animal *in-Vivo* Imaging System showed miR-520g upregulation promoted growth tumor xenograft growth in nude mice. Tumor volumes were measured by *In-Vivo* Imaging System weekly. After four weeks, xenograft weight and volume curves were compared with controls (*n* = 5, *p < 0.05, **p < 0.001; Left, miR-520g overexpression; Right, vector control). (**F**) Cell cycle analysis using FACS. Overexpression or knockdown of miR-520g induced or inhibited the G1 to S phase transition, respectively (*p* < 0.001). (**G**) Altered cell cycle-related proteins after miR-520g overexpression or knockdown *in vitro*. (**H**) IHC staining analysis for Ki-67, c-myc, cyclinD1 and PCNA in the miR-520g overexpression and control groups in tumor xenografts.

To investigate the underlying biological functions of miR-520g in EOC, we evaluated miR-520g expression in eight EOC cell lines. We selected A2780 and SKOV3 cell lines with low miR-520g expression, and MCAS and OVK18 cell lines with high miR-520g expression for further studies (Figure 2A). We developed stable, high miR-520g-expressing A2780 and SKOV3 cell lines and knocked down miR-520g expression in MCAS and OVK18 cells (Figure 2B and 2C). The CCK8 assay showed that ectopic overexpression or knockdown of miR-520g significantly increased or inhibited EOC cell growth *in vitro*, respectively (*p* < 0.05 for all, Figure 2D). Furthermore, *in vivo* experiments also revealed that overexpression or downregulation of miR-520g generated larger or smaller subcutaneous xenografts in nude mice, respectively, as compared to the control (*p* < 0.05 for both, Figure 2E and S1A). Both the *in vitro* and *in vivo* assays demonstrated that miR-520g accelerated EOC cell proliferation.

Cell cycle analysis was performed using fluorescence-activated cell sorting (FACS). We found that aberrant high miR-520g expression increased the percentage of S phase cells and decreased that of G0/G1 phase cells, whereas low miR-520g expression had the opposite effect (*p* < 0.05 for all, Figure 2F). These results were supported by altered cell cycle-related protein expression. *In vitro*, ectopic overexpression or knockdown of miR-520g resulted in increased or decreased expression of CDK4, CDK6, c-myc, and CyclinD1, respectively, which are indicators of cell proliferation and transition from G0/G1 to S phase (Figure 2G) [21]. Immunohistochemical (IHC) staining also confirmed that tumor xenografts with miR-520g overexpression or downregulation showed higher or lower expression of Ki-67, c-myc, CyclinD1 and PCNA, respectively, than controls (Figure 2H and S1B). These data suggest that miR-520g enhanced EOC cell proliferation by inducing the G0/G1 to S phase transition.

**Figure 3:**
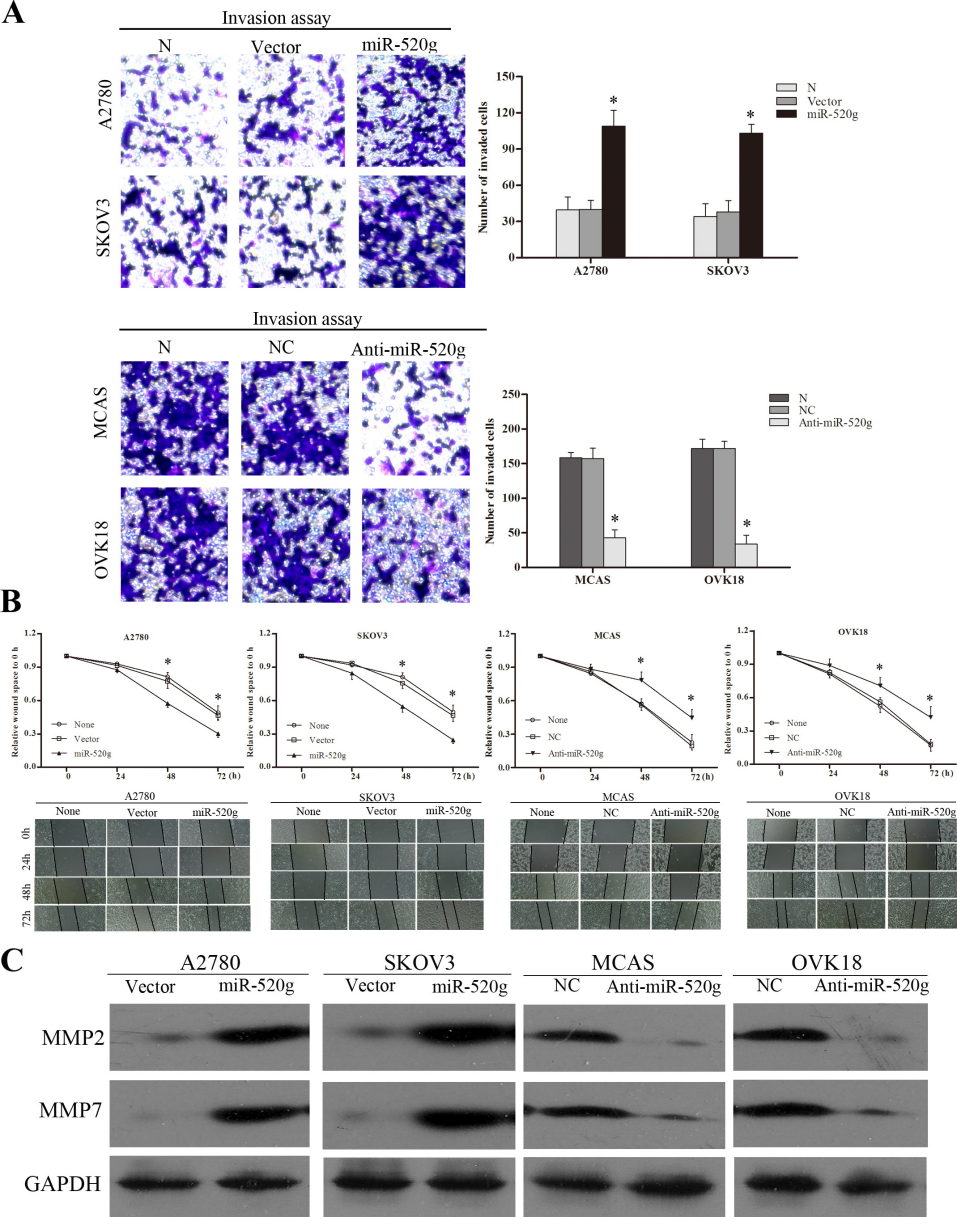
miR-520g enhanced EOC cell invasion and wound healing (**A**) miR-520g upregulation or downregulation increased or reduced invasive EOC cells, respectively (*p < 0.05). (**B**) Upregulation or downregulation of miR-520g accelerated or delayed wound healing, respectively (*p < 0.05). (**C**) Western blot analyses of MMP2 and MMP7 levels in miR-520g overexpressing and knocked down EOC cells.

Clinical analysis revealed that miR-520g expression was positively related to EOC differentiation and metastasis. Transwell invasion assays showed that overexpression or knockdown of miR-520g strengthened or attenuated EOC cell invasion, respectively (*p* < 0.05 for all, Figure 3A). Wound healing assays showed that overexpression or knockdown of miR-520g accelerated or delayed wound healing, respectively (*p* < 0.05 for all, Figure 3B). Western blotting demonstrated that levels of the tumor invasion and metastasis-associated proteins, MMP2 and MMP7, were increased or decreased in miR-520g overexpressing or downregulated EOC cells, respectively (Figure 3C). These results show that miR-520g promoted EOC cell invasion *in vitro*.

**Figure 4:**
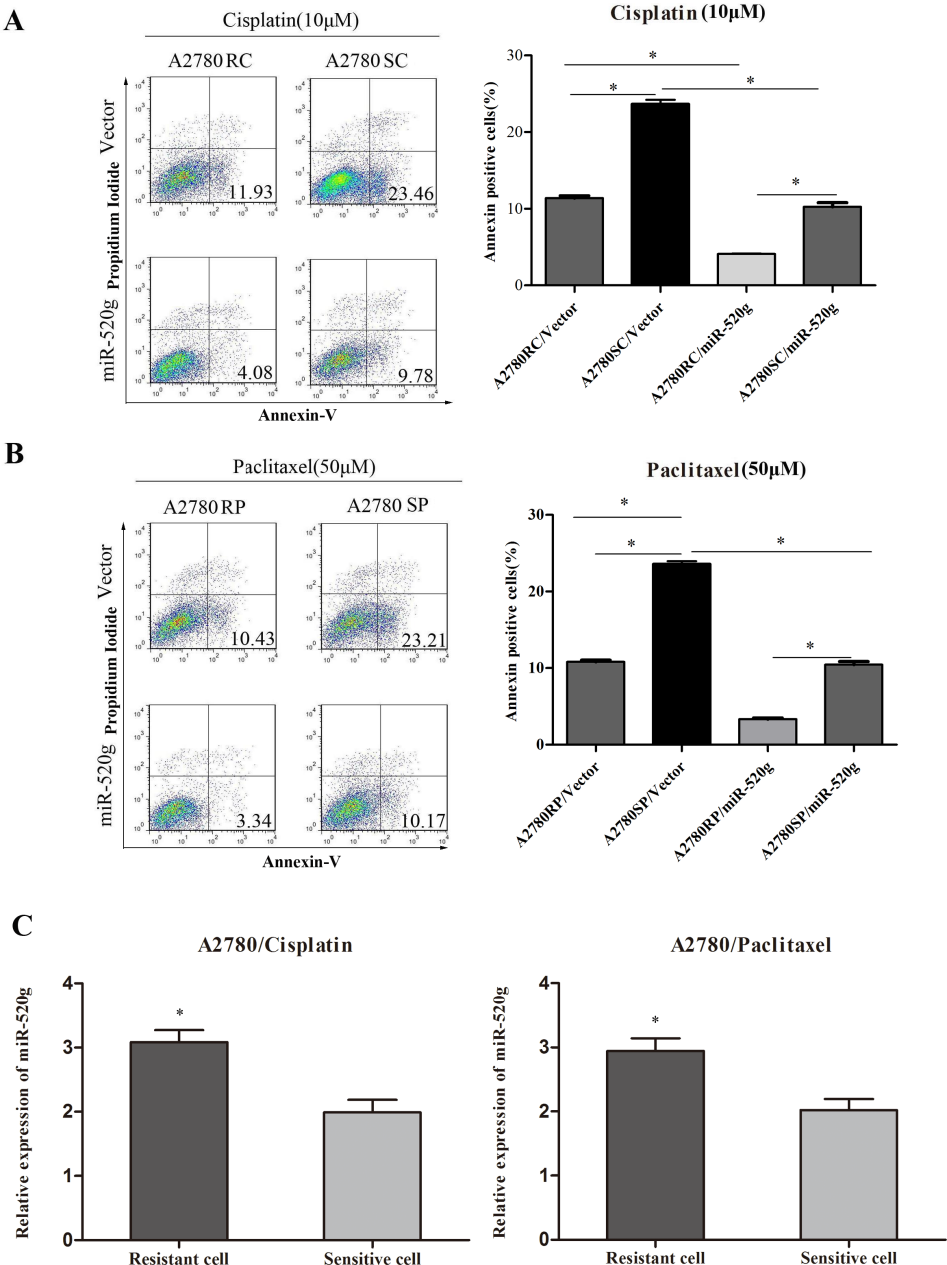
miR-520g increased chemoresistance to cisplatin and paclitaxel (**A**) Annexin V/PI staining followed by FACS analysis showed that miR-520g overexpression in A2780 cisplatin resistant (A2780RC) and sensitive (A2780SC) cells increased chemoresistance to cisplatin and inhibited apoptosis (*p < 0.05). (**B**) miR-520g overexpression in A2780 paclitaxel resistant (A2780RP) and sensitive (A2780SP) cells increased chemoresistance to paclitaxel and inhibited apoptosis (*p < 0.05). (**C**) miR-520g levels in A2780 cisplatin and paclitaxel resistant/sensitive cell lines (*p < 0.05).

To further investigate the role of miR-520g in chemoresistance, we produced paclitaxel and cisplatin resistant A2780 cells by treating cultures with gradually increasing concentrations of the drugs (data not shown). After overexpressing miR-520g in these cells, we added paclitaxel (50 μM) and cisplatin (10 μM) for 36 h, then analyzed cell apoptosis by Annexin V/PI staining and FACS. miR-520g overexpression reduced apoptosis rates induced by paclitaxel and cisplatin in both the resistant and sensitive cells, but apoptosis rates were higher in sensitive cells (Figure 4A and 4B, *p < 0.05). We found that miR-520g levels in paclitaxel and cisplatin resistant cells were significantly higher than in sensitive cells (Figure 4C, **p < 0.001). These results suggested that miR-520g promotes chemoresistance to platinum-based chemotherapy in EOC.

### DAPK2 is a direct target of miR-520g

**Figure 5:**
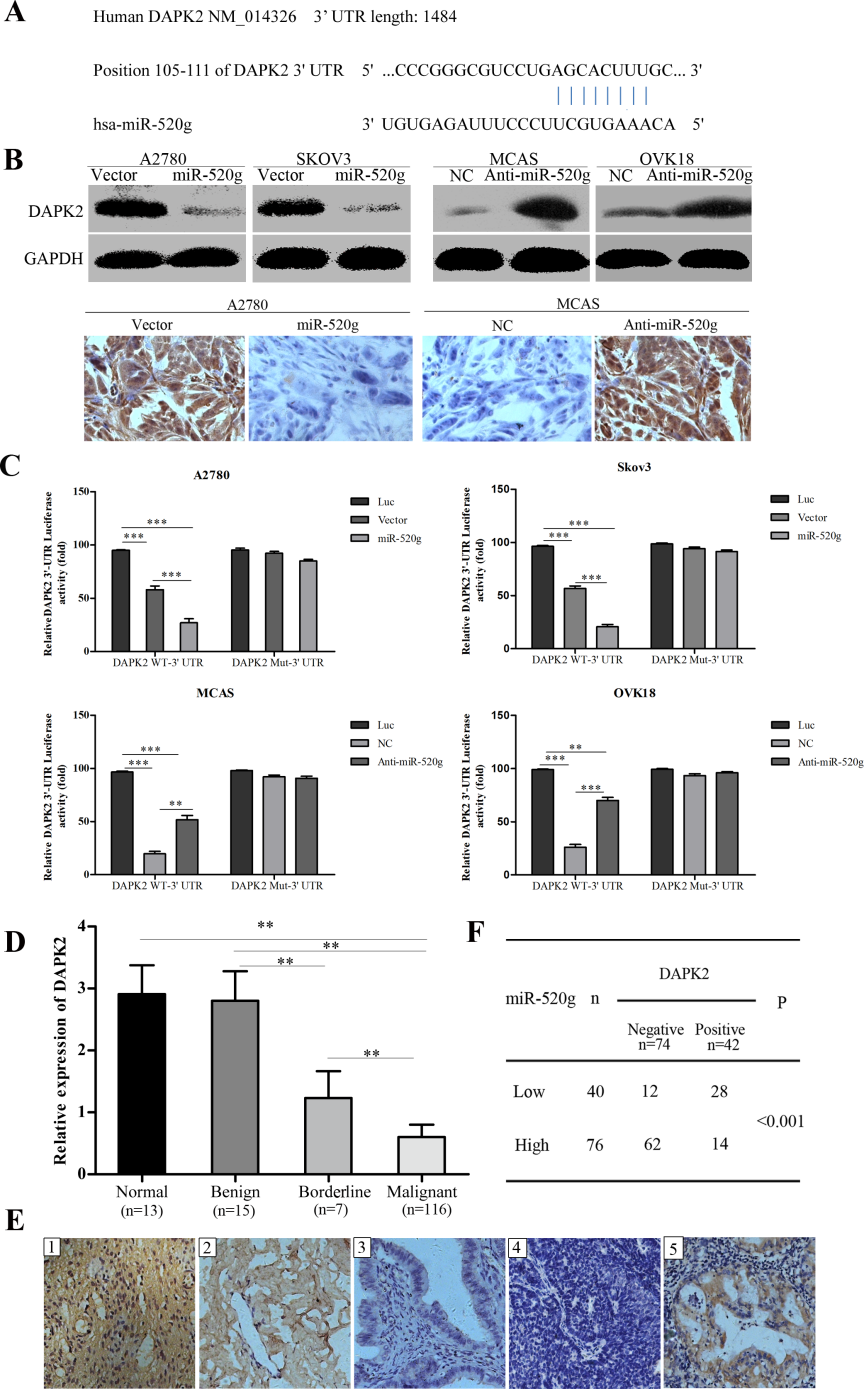
miR-520g directly targets DAPK2 in EOC cells and miR-520g expression was inversely associated with DAPK2 in EOC tissues (**A**) TargetScan predictions showed that miR-520g directly binds DAPK2. (**B**) Western blotting and tumor xenograft IHC staining showed that miR-520g inhibited DAPK2 post-transcriptionally. (**C**) Luciferase-dual reporter activity assay showed that miR-520g repressed DAPK2 expression in wild type but not mutant EOC cells. Luciferase-dual reporter vector (Luc) was the control (*p < 0.05, **p < 0.001, ***p < 0.0001). (**D**) Relative DAPK2 mRNA expression in normal (*n* = 13), benign (*n* = 15), borderline (*n* = 7) and EOC (*n* = 116) tissues (**p < 0.001). (**E**) DAPK2 IHC staining in normal, benign, borderline and EOC tissues: E1, strong DAPK2 staining in normal tissue; E2, moderate intensity staining in benign tissue; E3, weak intensity staining in borderline tissue; E4, negative staining in EOC tissue with high miR-520g expression; E5, strong staining in EOC tissue with low miR-520g expression (magnification × 200). (**F**) miR-520g was negatively correlated with DAPK2 expression in EOC tissues (**p < 0.001).

We used a bioinformatics approach involving the public TargetScan6.2 algorithm to identify the gene targets of miR-520g. We screened proliferation, cell cycle and tumor invasion-related targets whose 3′UTRs included the miR-520g-specific binding site, and identified DAPK2 as a candidate for further study (Figure 5A). DAPK2 is a tumor suppressor that promotes apoptosis and tumor cell death [22]. We compared the protein levels of DAPK2 in miR-520g overexpressing or downregulated cell lines using western blotting, and in tumor xenografts by IHC staining. We found that miR-520g overexpression or knockdown induced significant downregulation or upregulation of DAPK2 protein levels, respectively, in both EOC cell lines and tumor xenografts (Figure 5B). These results indicated that miR-520g negatively regulated DAPK2 expression post-transcriptionally.

To determine whether miR-520g directly regulated DAPK2 expression via 3′UTR degradation, we constructed luciferase-dual reporter plasmids with the 3′UTR of DAPK2 (wild type, WT or mutated, Mut) and co-transfected cells with miR-520g, anti-miR-520g or their correspondent negative controls. We found that miR-520g or anti-miR-520g only decreased or increased luciferase activity, respectively, when co-transfected with DAPK2 WT-3′UTR, but not with DAPK2 Mut-3′UTR (Figure 5C, **p < 0.001, ***p < 0.0001). Notably, luciferase activity resulting from miR-520g overexpression or knockdown was reversed by mutating the miR-520g-DAPK2 binding sites (Figure 5C). These results confirmed that DAPK2 is directly regulated by miR-520g in EOC cells.

We evaluated DAPK2 mRNA levels in 13 normal, 15 benign, 7 borderline and 116 EOC tissue samples, and found that DAPK2 expression gradually decreased from the normal to EOC tissue (*p* < 0.001 for all, Figure 5D). IHC staining showed DAPK2 in most of the low miR-520g-expressing tissues, including normal (Figure 5E1), benign (Figure 5E2) and borderline tissue (Figure 5E3), as well as the low miR-520g-expressing EOC tissues (Figure 5E5). By contrast, no DAPK2 staining was observed in the majority of EOC tissues with high miR-520g expression (Figure 5E4). There were 74 cases of DAPK2 negative staining and 42 of DAPK2 positive staining among 116 EOC tissues, which was negatively correlated with miR-520g expression (*p* < 0.001, Figure 5F).

### miR-520g directly suppresses DAPK2 causing proliferation, invasion and chemoresistance in EOC cells

**Figure 6:**
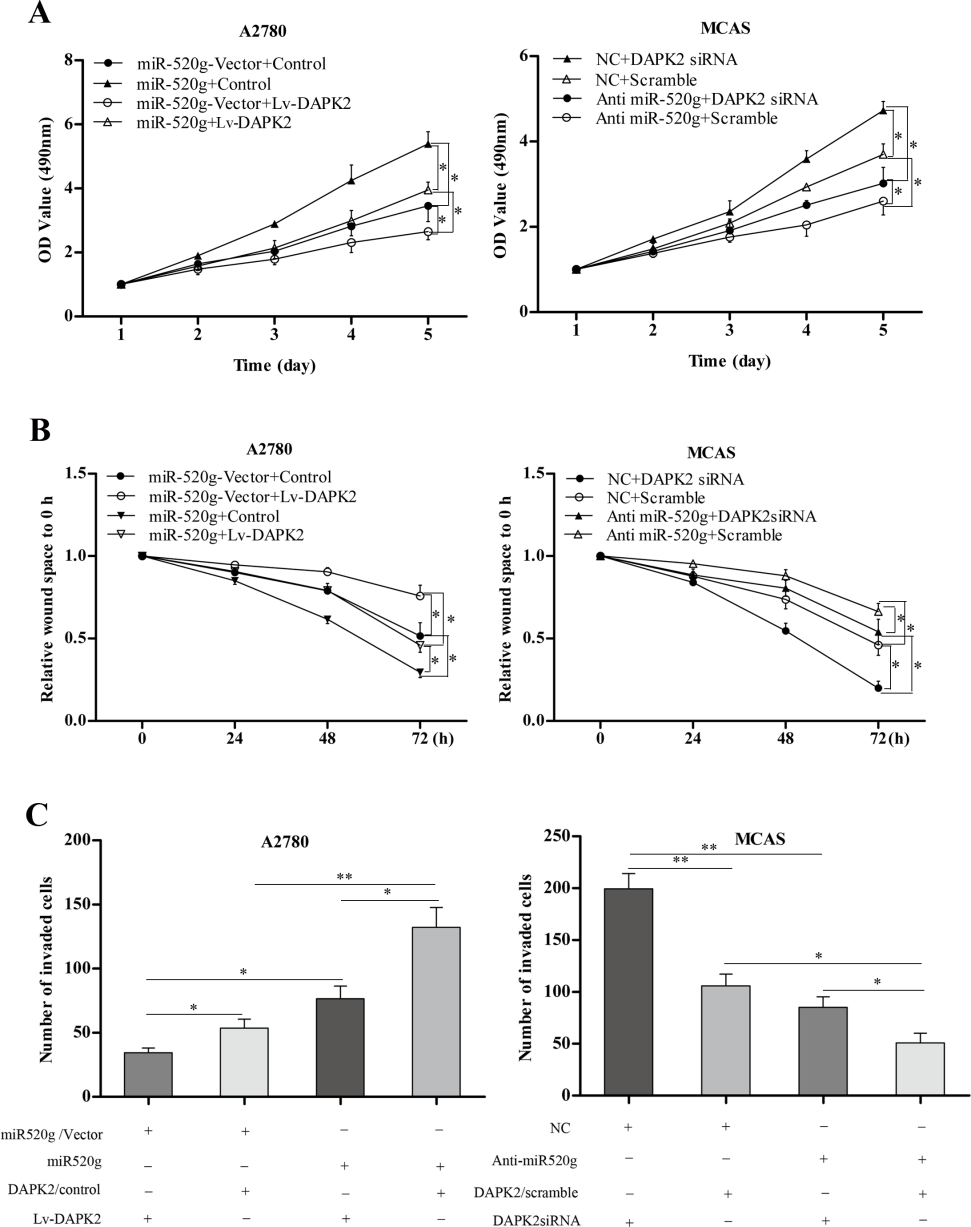
Role of DAPK2 in miR-520g-mediated tumor promotion (**A**) DAPK2 overexpression or knockdown partially reversed increased or decreased proliferation resulting from miR-520g upregulation or downregulation, respectively (*p < 0.05). (**B**) DAPK2 overexpression or knockdown partially neutralized increased or delayed wound healing induced by miR-520g upregulation or downregulation, respectively (*p < 0.05). (**C**) Overexpression or knockdown of DAPK2 partially abrogated increased or reduced invasion due to miR-20g upregulation or downregulation, respectively (*p < 0.05, **p < 0.001).

*In vitro* assays showed that DAPK2 inhibited tumor cell proliferation, wound healing and invasion. Additionally, miR-520g-promoted cell proliferation, wound healing and invasion was partially reversed by DAPK2 overexpression (Figure 6A–6C *p* < 0.05 for all). These results suggested that miR-520g-enhanced EOC progression is due, at least in part, to DAPK2 downregulation.

**Figure 7:**
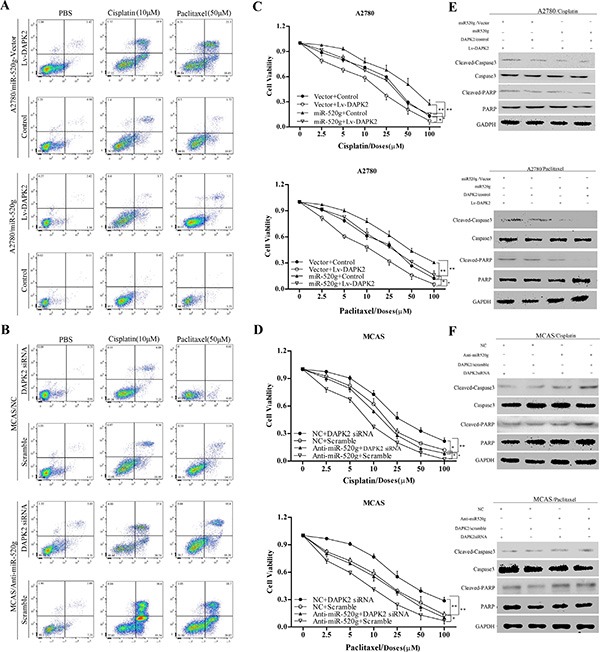
miR-520g increased chemoresistance to cisplatinum and paclitaxel and inhibited EOC cell viability by repressing DAPK2 (**A**) A2780/vector cells and A2780/miR-520g cells transfected with the Lv-DAPK2 or control vectors, respectively, were treated with PBS, cisplatin (10 μl) and paclitaxel (50 μl) for 36 hours. Apoptosis. Was analyzed using Annexin V/PI staining and FACS. DAPK2 overexpression decreased chemoresistance induced by miR-520g and increased sensitivity to cisplatin and paclitaxel. (**B**) MCAS/NC cells and MCAS/Anti-miR-520g cells were transfected with DAPK2/siRNA or scramble, respectively, and then treated with PBS, cisplatin (10 μl) and paclitaxel (50 μl) for 36 hours. Apoptosis. Was analyzed using Annexin V/PI staining and FACS. DAPK2 knockdown partially inhibited chemosensitivity induced by miR-520g knockdown. (**C**) After transfection with Lv-DAPK2 or control vectors, A2780/miR-520g-vector cells and A2780/miR-520g cells were treated with cisplatin and paclitaxel for 36 h. DAPK2 overexpression decreased cell viability promoted by miR-520g as measured by CCK8 assay (*p < 0.05, **p < 0.001). (**D**) Following transfection with DAPK2siRNA or scramble, MCAS/NC cells and MCAS/Anti-miR-520g cells were treated with cisplatin and paclitaxel for 36 h. DAPK2 knockdown partially restored cell viability (*p < 0.05, **p < 0.001). (**E**) Western blot analysis for caspase-3 and poly (ADP-ribose) polymerase (PARP) in A2780/vector cells and A2780/miR-520g cells transfected with the Lv-DAPK2 or control vectors, respectively, and treated with PBS, cisplatin (10 μl) and paclitaxel (50 μl) for 36 hours. (**F**) Western blot analysis for caspase-3 and PARP in MCAS/NC cells and MCAS/Anti-miR-520g cells transfected with the DAPK2/siRNA or scramble, respectively, and treated with PBS, cisplatin (10 μl) and paclitaxel (50 μl) for 36 hours.

Subsequently, we explored the role of DAPK2 in miR-520g-mediated cisplatin and paclitaxel chemoresistance. We overexpressed or knocked down DAPK2 in the miR-520g-overexpressing or knocked down EOC cell lines, and treated these cells with PBS, cisplatin (10 μM) and paclitaxel (50 μM). After 36 h, we detected apoptotic cells using Annexin V/PI staining and FACS. We found that chemoresistance cisplatin and paclitaxel induced by miR-520g was partially eliminated by DAPK2 overexpression (Figure 7A) Similarly, DAPK2 knockdown partially reversed chemosensitivity induced by miR-520g downregulation (Figure 7B). These results indicated that DAPK2 suppression contributes to miR-520g-meditated resistance to apoptosis and platinum-based chemotherapy in EOC cells.

**Figure 8:**
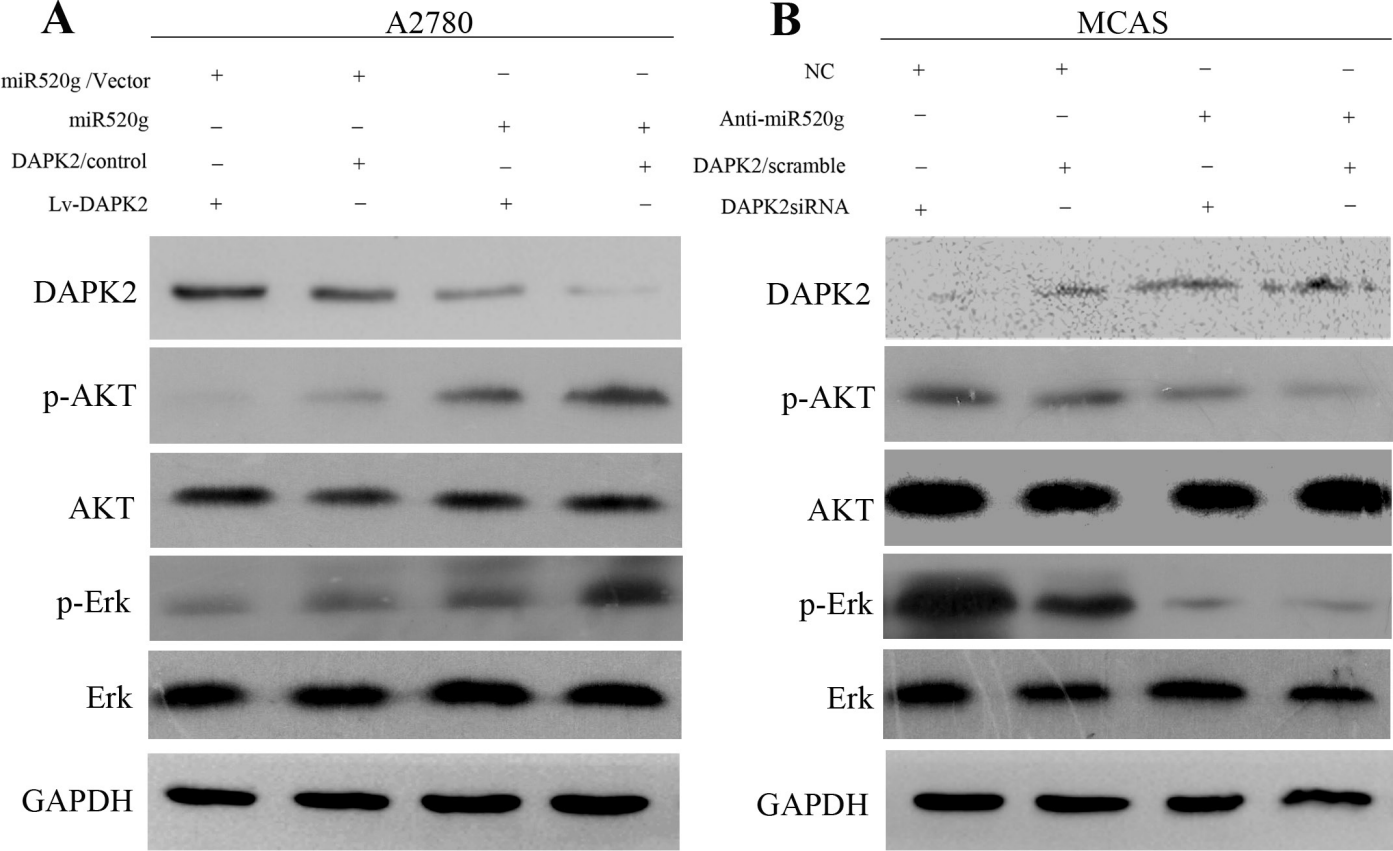
MAPK and AKT pathways promote miR-520g-DAPK2-mediated EOC cell proliferation (**A**) Western blot analyses of DAPK2, p-AKT, AKT, p-Erk and Erk in A2780/vector cells and A2780/miR-520g cells transfected with the Lv-DAPK2 or control vectors, respectively. (**B**) Western blot analyses of DAPK2, p-AKT, AKT, p-Erk and Erk in MCAS/NC cells and MCAS/Anti-miR-520g cells transfected with DAPK2/siRNA or scramble, respectively.

In addition, CCK8 assay results showed that miR-520g overexpression or knockdown significantly enhanced or weakened ovarian cancer cell viability, respectively, compared with controls. DAPK2 overexpression or knockdown partially abrogated these effects (Figure 7C and 7D), suggesting that DAPK2 suppression is important in miR-520g-enhanced EOC cell viability. Levels of proteins involved in apoptosis, such as caspase3 and PARP, were measured through western blotting. High miR-520g expression inhibited caspase3 and PARP activation, while DAPK2 overexpression partially rescued activation (Figure 7E and 7F). Because DAPK2 mainly induces apoptosis through MAPK and AKT signaling [23–25], we also evaluated the effect of miR-520g overexpression or knockdown on the activation of these pathways. We found that miR-520g overexpression or downregulation could increase or reduce the protein levels of p-AKT and p-Erk, respectively, while DAPK2 overexpression or knockdown could partially reverse these effects (Figure 8A and 8B). These results suggest that miR-520g may activate the MAPK and AKT pathways and enhance EOC cell chemoresistance and viability by repressing DAPK2.

## DISCUSSION

While tumor progression and chemoresistance are usually associated with poor prognosis and reduced survival in EOC patients, the mechanisms underlying these traits are still not fully determined [26–28]. Mounting evidence suggests that miRNAs play a significant role in EOC tumorigenesis and development [6, 29–31]. In the present study, we found that high miR-520g expression promoted EOC progression, and that miR-520g upregulation increased chemoresistance to platinum-based chemotherapy. These findings highlight the important role of miR-520g in predicting prognosis and chemosensitivity to platinum-based chemotherapy in EOC patients. Further, these results indicate that miR-520g may be a therapeutic target for EOC treatment, and stronger postoperative chemotherapy regimens are indicated for patients with high miR-520g expression.

High cell proliferation and invasion rates play important roles in EOC progression [32, 33]. To investigate the underlying biological functions of miR-520g in EOC, we firstly evaluated the expression of miR-520g in 8 EOC cell lines and selected A2780 and SKOV3 cell lines with low miR-520g expression and MCAS and OVK18 cell lines with high miR-520g expression for further studies. These different expression levels of miR-520g in 8 EOC cell lines might be correlated with the cell lines different abilities of proliferation rate, invasiveness and chemoresistance. We demonstrated *in vitro* and *in vivo* that miR-520g regulated the expression of proliferation and cell cycle-associated proteins and promoted EOC cell proliferation by inducing the G1 to S phase transition. A previous study reported that high miR-520g expression promoted HCC invasion and metastasis by inducing EMT [16]. In addition, miR-520g also induced endometriosis via upregulation of MMP2 expression, which is an indicator of tumor metastasis [34, 35]. Consistent with these reports, we found that miR-520g upregulation enhanced migration and invasion in EOC as measured by wound healing and transwell migration and invasion assays.

Loss or downregulation of tumor suppressors plays a critical role in tumorigenesis, progression and resistance to chemotherapy [31, 36]. Further, onco-miRNAs act by downregulating tumor suppressors [37, 38]. miR-520 h, a member of the miR-520 family, reportedly downregulates tumor suppressor DAPK2 expression and contributes to chemoresistance in breast cancer [39]. miR-520 h directly inhibited DAPK2 expression, and DAPK2 suppression was important in miR-520 h-mediated paclitaxel resistance in breast cancer cells. In the same study, restoring DAPK2 expression abolished miR-520 h-mediated tumor promotion and drug resistance [39]. DAPK2 mainly induces apoptosis through the extracellular signal-regulated kinase (ERK), mammalian target of rapamycin complex 1 (mTORC1), mitogen-activated protein kinases (MAPK) and AKT signal pathways [23–25]. In human obesity, DAPK2 knockdown led to decreased adipocyte autophagic clearance [41]. In human uterine and ovarian cancer tissues, DAPK expression was reportedly downregulated or methylation of the DAPK promoter was increased [19]. In addition, DAPK downregulation attenuated irinotecan-induced ovarian follicular apoptosis [42]. These previous studies suggest that DAPK2 loss or downregulation may be important in EOC progression and chemoresistance.

The results of the current study suggested that miR-520g downregulates DAPK2 expression by recognizing and binding to the DAPK2 3′UTR, and degradation of DAPK2 via targeting by miR-520g was confirmed by luciferase-dual activity reporter assay in EOC cells. In addition, DAPK2 was negatively associated with miR-520g expression in EOC tissues. Further, miR-520g-induced proliferation, migration and invasion were partially abrogated by DAPK2 overexpression. As miR-520 h and miR-520g both are the member of the miR-520 family, and they can both down regulate DAPK2 expression in cancer. Whether the effect of miR-520g on DAPK2 is specific or all the members of miR-520 family have the same effect of DAPK2 in ovarian cancer is worth to be further explored.

miR-520g reportedly confers resistance to 5-FU in colorectal cancer [17]. Here, miR-520g was also involved in chemoresistance to cisplatin or paclitaxel, and miR-520g expression levels in the cisplatin or paclitaxel resistant A2780 cells were significantly higher than in sensitive A2780 cells. Chemoresistance associated with miR-520g was antagonized by DAPK2, which was confirmed by apoptosis and cell viability assays. We also demonstrated miR-520g could activate the MAPK and AKT pathways by repressing DAPK2 in EOC cells.

In conclusion, our findings suggest that miR-520g promotes EOC progression and chemoresistance by negatively regulating DAPK2 expression. Notably, chemoresistant patients with high miR-520g expression also showed low DAPK2 expression. miR-520g and DAPK2 together may be valuable prognostic markers and could be useful for identifying EOC patients at high risk for resistance to platinum-based chemotherapy.

## MATERIALS AND METHODS

### Patients and tissues samples

We obtained 13 normal, 15 benign, 7 borderline and 116 EOC tissue samples from the Department of Gynecology and Obstetrics, Shanghai Jiaotong University Affiliated First Peoples' Hospital. All patients were clinically and histopathologically diagnosed between June 2008 and June 2009. Based on chemosensitivity to therapy and previous study criteria, we divided the 116 EOC patients into two groups: the chemosensitive group and the chemoresistant group [43]. The 116 EOC patients' clinicopathological data are summarized in Table 1. No cancer patient received neoadjuvant anticancer therapy. All cancer patients were treated with platinum-based chemotherapy (TP or PAC) post-surgery (Table S1). We obtained written and informed consent from all patients. This study was approved by the Institutional Research Ethics Committee of Shanghai Jiaotong University Affiliated First Peoples' Hospital.

### Cell lines and cultures

The eight human EOC cell lines used in this study were: A2780, SKOV3, OVA433, ES-2, OV2008, CaOV-3, MCAS, and OVK18, and were purchased from the American Type Culture Collection. All cell lines were cultured in RPMI-1640 medium (GIBCO, Carlsbad, CA, USA) with 10% fetal bovine serum (FBS; GIBCO) at 5% CO2 in a 37°C humidified atmosphere.

### RNA extraction and real-time quantitative PCR

Total RNA was extracted from cell lines and paraffinized tissues using Trizol reagent (Invitrogen, Carlsbad, CA, USA) and the High Pure FFPE RNA Micro Kit (Roche, Basle Switzerland) according to the manufacturer's instructions. After synthesizing cDNA, miR-520g was amplified using real-time PCR, with the TaqMan Human miRNAs qPCR Quantitation Kit (Applied Biosystems, Foster City, CA, USA) used for quantification following amplification. The small nuclear RNA U6 was used as control. The Quantitative SYBR Green PCR Kit (Qiagen, Hilden, Germany) was used to quantify DAPK2 mRNA using GAPDH as the internal control. The primers used in this study were as reported previously [16]. All experiments were performed in at least triplicate.

### Transfection with overexpression vectors, plasmids and anti-miR

The miR-520g and DAPK2 vectors, including overexpression vectors, knockdown vectors and the corresponding negative controls, were constructed as previously reported [16, 36]. The constructed vectors were transfected into selected cells using Lipofectamine 2000 according to the manufacturer's instructions (Invitrogen).

### Western blot analysis and immunohistochemistry

Western blotting and immunohistochemical staining were performed to analyze the protein levels of DAPK2 and cell cycle and proliferation-related genes as previously reported [44]. The percentage of positive cells was scored as follows: 1) staining area score: 0′, < 10%; 1′, 10–30%; 2′, 31–50%; 3′, > 50%; 2) staining intensity score: 0′, no staining; 1′, mild staining; 2′, moderate staining; 3′, intense staining; 3) total staining score based on both staining area and intensity: 0–2′, negative expression; 3–4′, medium expression; 5–6′, strong expression. Table S2 describes all antibodies used in this work.

### Cell proliferation and viability assay *in vitro*

Two thousand EOC cells per well were plated in 96-well plates and treated with or without PBS, Cisplatinum and paclitaxel. After 36 h, *Cell Counting Kit-8* (CCK8 Beyotime, Biotechnology, Jiangsu, China) was used to measure the 490 nm OD values of each well at multiple time points using a Sunrise Micro plate Reader (Tecan, Mannedorf, Switzerland).

### Transwell and wound healing assays

In the transwell migration and invasion assay, 1 × 10^5^ cells/100 μL were seeded in the upper chambers of 24-well plates (Corning, NY, USA) coated with BD matrigel basement membrane matrix with serum-free medium. DMEM/F12 supplemented with 10% FBS was added to the lower chamber. After 24 h, cells on the upper side of the chamber were removed, and cells on the lower side were fixed with formaldehyde and stained with crystal violet. The invasive cells were counted under a microscope.

The wound healing experiment was performed by plating 1 × 10^5^ cells per well in 6-well plates, and scraping a wound when the cells reached the exponential growth phase. Images were captured at 0, 24, 48 and 72-h intervals, and wound widths were quantified and compared to baseline values.

### Flow cytometry

During cell cycle analyses, cells in the logarithmic growth phase were washed three times with PBS. Suspended cells at a concentration of 1 × 10^6^/ml were fixed with 70% ethanol for 0.5 h. Following propidium iodide (PI) staining, DNA content was measured using flow cytometry (BD Biosciences, San Jose, CA, USA).

The cellular apoptosis assay was performed using the PI/Annexin V-APC Apoptosis Kit (Sigma) according to the manufacturer's instructions. A FACScan flow cytometer and FlowJo software (Tree Star Inc., Ashland, OR) were used to analyze the staining data. All experiments were repeated in triplicate.

### Construction of luciferase reporters and activity assay

The 1484-bp 3′UTR of the DAPK2 gene (DAPK2 WT-3′UTR) with the specific miR-520g binding sequence was amplified and inserted into the Dual-Luciferase Reporter vector, and co-transfected with a miR-520g mimic into the selected ovarian cancer cells using Lipofectamine 2000 according to the manufacturer's instructions. TK-Renilla plasmid was used as the control. miR-520g with a mutant DAPK2 3′UTR site (AAA to CCC, Mut-miR-520g) and DAPK2 WT-3′UTR with a mutant miR-520g binding site (TTT to GGG, DAPK2 Mut-3′UTR, inserted into the Dual-Luciferase Reporter vector) were similarly transfected into selected cell lines. After transfection for 48 h, cell lysate was collected using the Dual-Luciferase Reporter Assay Kit (Promega). A micro plate luminescence counter (Perkin Elmer) was used to detect luciferase activity.

### Nude mouse xenografted tumor model

The *in vivo* assay using nude mice was approved by the Institutional Animal Care and Use Committee of Shanghai Jiaotong University Affiliated First People's Hospital. Four-week-old male, specific pathogen-free BALB/C nude mice were purchased from Shanghai Research Center for Model Organisms. We subcutaneously injected 1 × 10^7^A2780 cells transfected with miR-520g or vector in 200 μL RPMI-1640 medium into the left or right flanks of nude mice, respectively. Tumor volumes were measured weekly using an *In-Vivo* Imaging System (IVIS; Xenogen). After four weeks, the mice were euthanized. Xenografted tumor tissue samples were obtained and embedded in paraffin.

### Statistical analysis

The significance of categorical variables was compared using Fisher's exact test or Chi-square test. Differences in continuous variables were analyzed using one-way analysis of variance or Student's *t*-test. Overall survival (OS) was evaluated by Kaplan-Meier analyses with log-rank test. The hazard ratio and 95% confidence intervals of OS were estimated by Cox proportional hazards model. *P* < 0.05 was considered statistically significant. Error bars in all bar graphs represent SD or SEM as specified. All data analyses were conducted using SPSS 17.0 statistical software (SPSS Inc., Chicago, IL).

## SUPPLEMENTARY MATERIALS FIGURES AND TABLES


