# Supplementary material for: MicroRNA-520g promotes epithelial ovarian cancer progression and chemoresistance via DAPK2 repression
Source: Oncotarget. 2016 Apr 1;7(18):26516–34. doi: 10.18632/oncotarget.8530 (PMC5041996; doi:10.18632/oncotarget.8530)
Supplement: Supplementary file 1 [file oncotarget-07-26516-s001.pdf]

MicroRNA-520g promotes epithelial ovarian cancer progression and chemoresistance via DAPK2 repression

Supplementary Materials

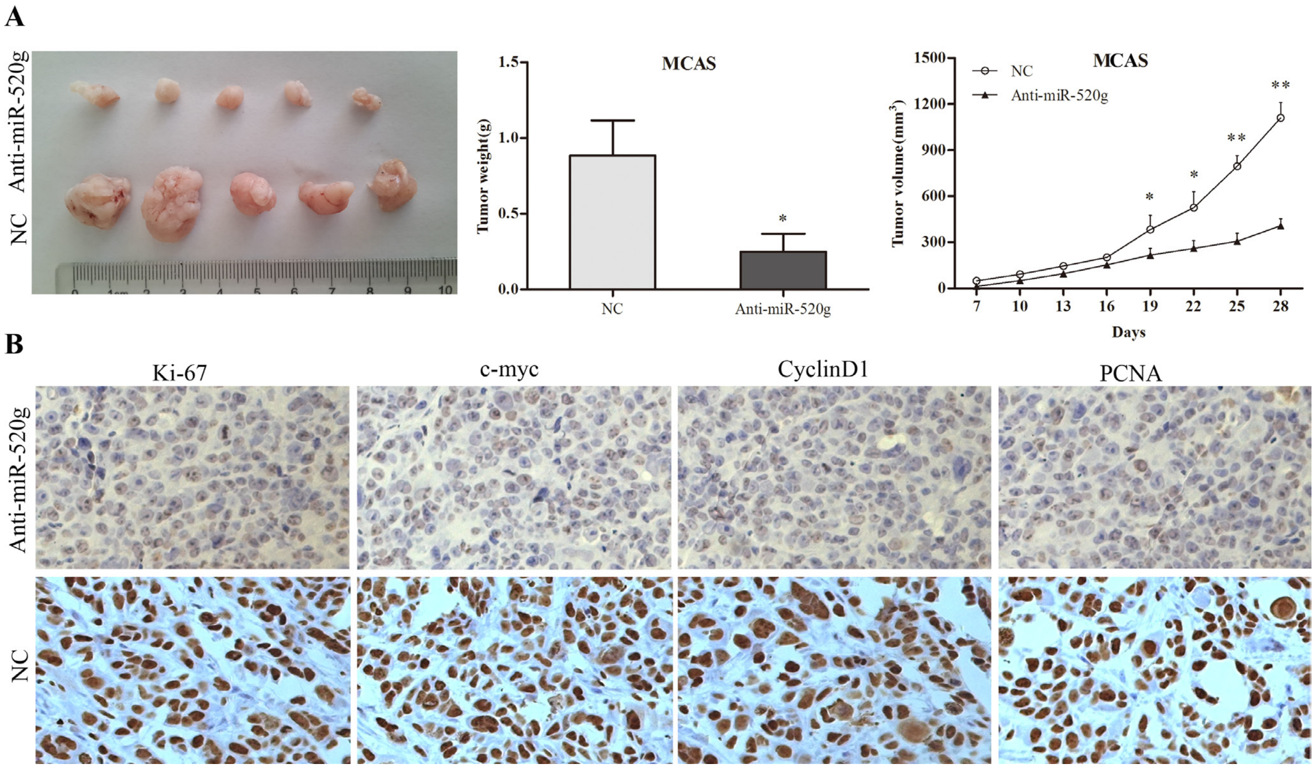

Supplementary Figure S1: miR-520g knockdown inhibited proliferation of MACS cells *in vivo*. (**A**) miR-520g knockdown in MACS cells via anti-miR-520g mimics inhibited the growth of subcutaneous xenografted tumors in nude mice. Tumor weight and volume curves are shown at four weeks (n = 5, *p < 0.05, **p < 0.001). (**B**) IHC staining of Ki-67, c-myc, CyclinD1 and PCNA in miR-520g-downregulated and control xenografted tumors (magnification × 200).

Supplementary Table S1: Postoperative chemotherapy plans for patients with EOC

Regimens	Dose	Administration	Time for drug	Interval cycles	cycles
**TP**					
Paclitaxel	175 mg/m^2	i.v. (2 h Inf)	Day1	Every 3 weeks	Six-week cycles
Cisplatin	75 mg/m^2	i.v.after paclitaexl 1 h			
**PAC**					
Cisplatin	70 mg/m^2	i.v. (one time)	Day1	Every 3 weeks	Six-week cycles
Cyclophosphamide	70 mg/m^2				
Epirubicin	50 mg/m^2				

Supplementary Table S2: Data of antibodies used in our research

Antibody	WB	IHC	Specificity	Company
DAPK2 (NBP2–02477)	1:1000	1:50	Rabbit monoclonal	Novus Biologicals
CyclinD1 (sc-246)	1:500	1:100	Mouse monoclonal	Santa Cruz Biotechnology
c-myc (BS2462)	1:500	1:200	Rabbit polyclonal	Biowold
CDK4 (sc-260)	1:500	-	Rabbit polyclonal	Santa Cruz Biotechnology
CDK6 (sc-7961)	1:500	-	Mouse monoclonal	Santa Cruz Biotechnology
Ki-67 (#12202)	-	1:100	Mouse monoclonal	Cell Signaling Technology
PCNA (ab18197)		1:100	Rabbit polyclonal	Abcam
GAPDH (ab181602)	1:10000	-	Rabbit monoclonal	Abcam
AKT (#4685)	1:1000		Rabbit monlclonal	Cell Signaling Technology
p-AKT (#4060)	1:2000		Rabbit monlclonal	Cell Signaling Technology
Erk (#4348)	1:1000		Rabbit monlclonal	Cell Signaling Technology
p-Erk (#4370)	1:2000		Rabbit monlclonal	Cell Signaling Technology
MMP2 (#13132)	1:1000		Rabbit monlclonal	Cell Signaling Technology
MMP7 (#3801)	1:1000		Rabbit monlclonal	Cell Signaling Technology
Caspase 3 (#9665)	1:1000		Rabbit monlclonal	Cell Signaling Technology
Cleaved Caspase3 (#9664)	1:1000		Rabbit monlclonal	Cell Signaling Technology
PARP (ab6079)	1:400		Rabbit monlclonal	Abcam
